# Classification of triple-negative breast cancers based on Immunogenomic profiling

**DOI:** 10.1186/s13046-018-1002-1

**Published:** 2018-12-29

**Authors:** Yin He, Zehang Jiang, Cai Chen, Xiaosheng Wang

**Affiliations:** 1 Biomedical Informatics Research Lab, School of Basic Medicine and Clinical Pharmacy, Nanjing, 211198 China; 2 Cancer Genomics Research Center, School of Basic Medicine and Clinical Pharmacy, Nanjing, 211198 China; 3 Big Data Research Institute, China Pharmaceutical University, Nanjing, 211198 China; 4 Department of Electrical and Computer Engineering, University of California, San Diego, La Jolla, CA 92093 USA

**Keywords:** Triple-negative breast cancer, Tumor immunity, Immunogenomic profiling, Classification, Machine learning

## Abstract

**Background:**

Abundant evidence shows that triple-negative breast cancer (TNBC) is heterogeneous, and many efforts have been devoted to identifying TNBC subtypes on the basis of genomic profiling. However, few studies have explored the classification of TNBC specifically based on immune signatures that may facilitate the optimal stratification of TNBC patients responsive to immunotherapy.

**Methods:**

Using four publicly available TNBC genomics datasets, we classified TNBC on the basis of the immunogenomic profiling of 29 immune signatures. Unsupervised and supervised machine learning methods were used to perform the classification.

**Results:**

We identified three TNBC subtypes that we named Immunity High (Immunity_H), Immunity Medium (Immunity_M), and Immunity Low (Immunity_L) and demonstrated that this classification was reliable and predictable by analyzing multiple different datasets. Immunity_H was characterized by greater immune cell infiltration and anti-tumor immune activities, as well as better survival prognosis compared to the other subtypes. Besides the immune signatures, some cancer-associated pathways were hyperactivated in Immunity_H, including apoptosis, calcium signaling, MAPK signaling, PI3K–Akt signaling, and RAS signaling. In contrast, Immunity_L presented depressed immune signatures and increased activation of cell cycle, Hippo signaling, DNA replication, mismatch repair, cell adhesion molecule binding, spliceosome, adherens junction function, pyrimidine metabolism, glycosylphosphatidylinositol (GPI)-anchor biosynthesis, and RNA polymerase pathways. Furthermore, we identified a gene co-expression subnetwork centered around five transcription factor (TF) genes (*CORO1A*, *STAT4*, *BCL11B*, *ZNF831*, and *EOMES)* specifically significant in the Immunity_H subtype and a subnetwork centered around two TF genes (*IRF8* and *SPI1*) characteristic of the Immunity_L subtype.

**Conclusions:**

The identification of TNBC subtypes based on immune signatures has potential clinical implications for TNBC treatment.

**Electronic supplementary material:**

The online version of this article (10.1186/s13046-018-1002-1) contains supplementary material, which is available to authorized users.

## Introduction

Triple-negative breast cancer (TNBC) is a breast cancer subtype that lacks the expression of hormone receptors (estrogen receptor (ER) and progesterone receptor (PR)) and human epidermal growth factor receptor 2 (HER2). TNBC is associated with a high risk of mortality for its aggressiveness and the lack of effective targeted therapies. Moreover, abundant evidence shows that TNBC is very heterogeneous [1–4]. Lehmann et al. identified six gene expression profile-based TNBC subtypes, including an immunomodulatory (IM) subtype that was enriched in immune cell processes [2]. Bonsang-Kitzis et al. identified six TNBC subgroups based on a biological network-driven approach, which included two immunity clusters whose stromal immune module gene signatures exhibited a strong prognostic value [3]. Burstein et al. identified four stable TNBC subgroups based on mRNA expression and DNA genomic profiling, which included Luminal/Androgen Receptor, Mesenchymal, Basal-Like Immune Suppressed, and Basal-Like Immune Activated (BLIA); furthermore, the authors identified potential therapeutic targets for these specific subtypes [4]. These efforts to classify TNBC might lay the foundation for developing targeted therapies for TNBC.

Recently, cancer immunotherapy has been successful in treating many refractory malignancies [5]. Thus, it is worth considering immunotherapy for TNBC, since the therapeutic options for this disease are significantly limited. Indeed, many experimental and clinical studies have explored the possibility of treating TNBC patients with immunotherapy [6–11]. Moreover, numerous studies have demonstrated that TNBC is more immunogenic than other breast cancer **(**BC) subtypes, which may warrant an immunotherapeutic approach for TNBC [12, 13]. However, currently, immunotherapeutic strategies exhibit beneficial effects in less than 20% of cancer patients. This suggests that not all TNBC patients could respond to immunotherapy. In fact, certain genetic or genomic features, such as tumor mutation burden (TMB), neoantigen load, PD-L1 expression, and deficient DNA mismatch repair, have been associated with cancer immunotherapeutic responsiveness [14–18].

In this study, we classified TNBC into three distinct subtypes by immunogenomic profiling: Immunity High (Immunity_H), Immunity Medium (Immunity_M**)**, and Immunity Low (Immunity_L). We demonstrated the stability and reproducibility of this classification in four independent datasets by a machine learning approach. Furthermore, we identified the subtype-specific molecular features, including genes, gene ontology, pathways, and networks. The identification of immune signature-associated TNBC subtypes may facilitate the optimal selection of TNBC patients responsive to immunotherapy.

## Methods

### Clustering

For each TNBC dataset, we first quantified the enrichment levels of the 29 immune signatures in each TNBC sample by the single-sample gene-set enrichment analysis (ssGSEA) score [19, 20]. Based on the enrichment levels (ssGSEA scores) of the 29 immune signatures, we performed hierarchical clustering of TNBC.

### Evaluation of immune cell infiltration level, tumor purity, and stromal content in TNBC

ESTIMATE [21] was used to evaluate the immune cell infiltration level (immune score), tumor purity, and stromal content (stromal score) for each TNBC sample.

### Gene-set enrichment analysis

We performed gene-set enrichment analysis of the METABRIC and TCGA datasets by GSEA (R implementation) [22–24]. This analysis identified the KEGG [25] pathways that were upregulated in Immunity_H and Immunity_L (FDR < 0.05), respectively. The common pathways identified in both datasets were selected.

### Correlation of pathway activities with immune cell infiltration levels in TNBC

We quantified the activity of a pathway with the ssGSEA score of the set of genes included in the pathway, and the immune cell infiltration level with the immune score. The Spearman correlation of the ssGSEA score and the immune score were used to evaluate the correlation of pathway activities with immune cell infiltration levels in TNBC.

### Identification of TNBC subtype-specific gene ontology and networks

We used WGCNA [26] to identify the gene modules (gene ontology) that were significantly associated with the genes highly correlated with immune cell infiltration based on gene co-expression analysis. The gene modules specifically amplified in different TNBC subtypes were identified. On the basis of the expression correlations between the hub genes in the gene modules, we built gene–gene interaction networks. A hub gene was defined as a gene that was connected to no less than 10 other genes, with a connectedness weight greater than 0.25.

### Survival analyses

We compared the survival prognosis (overall survival (OS), disease-free survival (DFS), and metastasis-free survival (MFS) of TNBC patients considering tumor subtype and the expression level of the identified genes, i.e., higher expression level (expression levels > median) versus lower expression level (expression levels < median). The log-rank test was used to calculate the significance of survival time differences using a threshold of *P-*value < 0.05. Kaplan–Meier curves were plotted to show the survival time differences. We performed the survival analyses using the METABRIC, TCGA, and GSE103091 datasets, where the survival data were available.

### Class prediction

We transformed each attribute (immune signature or gene set) value (ssGSEA score) *x*_*i*_ into *x*_*i*_′ by the equation *x*_*i*_′ = (*x*_*i*_ − *x*_min_)/(*x*_max_ − *x*_min_), where *x*_min_ and *x*_max_ represent the minimum and maximum of the ssGSEA scores for the gene set across all TNBC samples, respectively. The Random Forest (RF) classifier was used to classify the TNBC subtypes. We set the number of trees to 100 and all 29 immune signatures as features for the RF classifier. The classification performance was evaluated by the accuracy and the weighted F-score. We carried out the classification in Weka [27].

### Comparison of the proportions of immune cell subsets between TNBC subtypes

CIBERSORT [28] was used to calculate the proportions of 22 human immune cell subsets. We set 1000 permutations and *P* < 0.05 as the criteria for the successful deconvolution of a sample. We compared the proportions of the immune cell subsets between TNBC subtypes using the Mann–Whitney U test.

### Comparison of clonal heterogeneity between the TNBC subtypes

We used the ABSOLUTE algorithm [29] to assess the ploidy score, representing clonal heterogeneity, for each TNBC sample. We compared the ploidy scores between the TNBC subtypes using the Kruskal–Wallis test.

### Comparison of biological processes between the TNBC subtypes

We compared the activities (ssGSEA scores) of stem cell-associated (marker genes *ABCA8* and *ALDH1A1*), proliferation (*MKI67*), and epithelial-to-mesenchymal transition (EMT) (*ZEB1*, *ZEB2*, *SNAIL*, *CDH2* and *TGFB1*) biological processes between the TNBC subtypes. The Kruskal–Wallis test was used to determine the statistical significance of the results.

### Comparison of somatic copy number alteration (SCNA) levels between the TNBC subtypes

We applied GISTIC2 [30] to the SNP6 file of the SCNA data for TNBC in TCGA. We obtained arm-level SCNA frequencies for Immunity_H and Immunity_L TNBC samples and compared them. Moreover, we calculated focal SCNA levels for each TNBC samples and compared them between Immunity_H and Immunity_L.

## Results

### Immunogenomic profiling identifies three TNBC subtypes

**Fig. 1:**
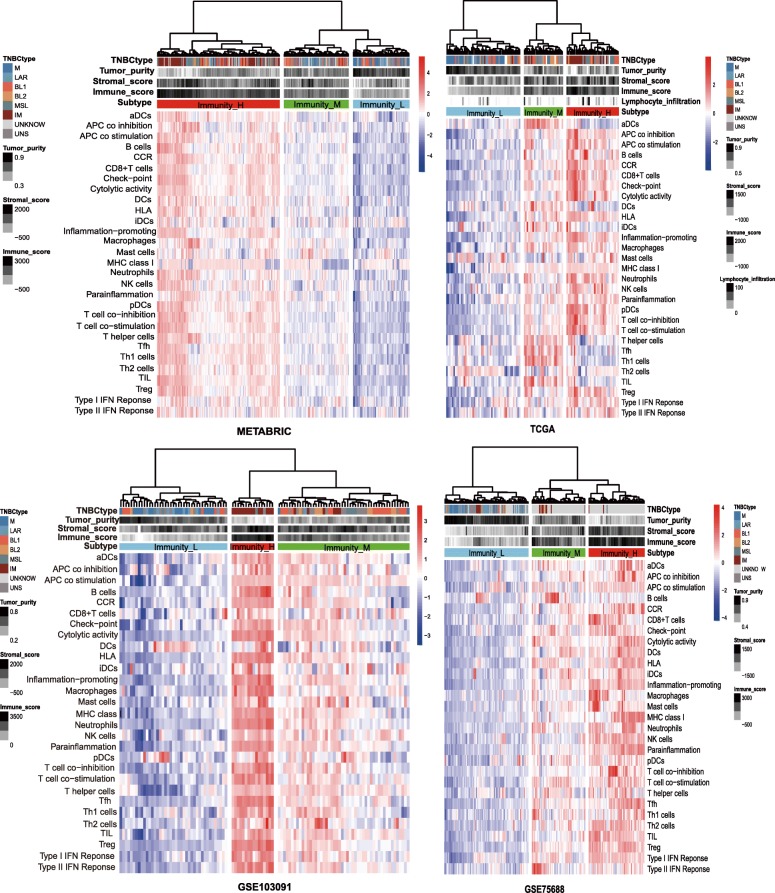
Hierarchical clustering of triple-negative breast cancer (TNBC) yields three stable subtypes in four different datasets. Immunity_H, Immunity High; Immunity_M, Immunity Medium; Immunity_L, Immunity Low. TNBCtype, a method for classifying TBNC [2, 37]. Tumor_purity, Stromal_score, and Immune_score were evaluated by ESTIMATE [21]. Lymphocyte_infiltration, percent of lymphocyte infiltration

**Fig. 2:**
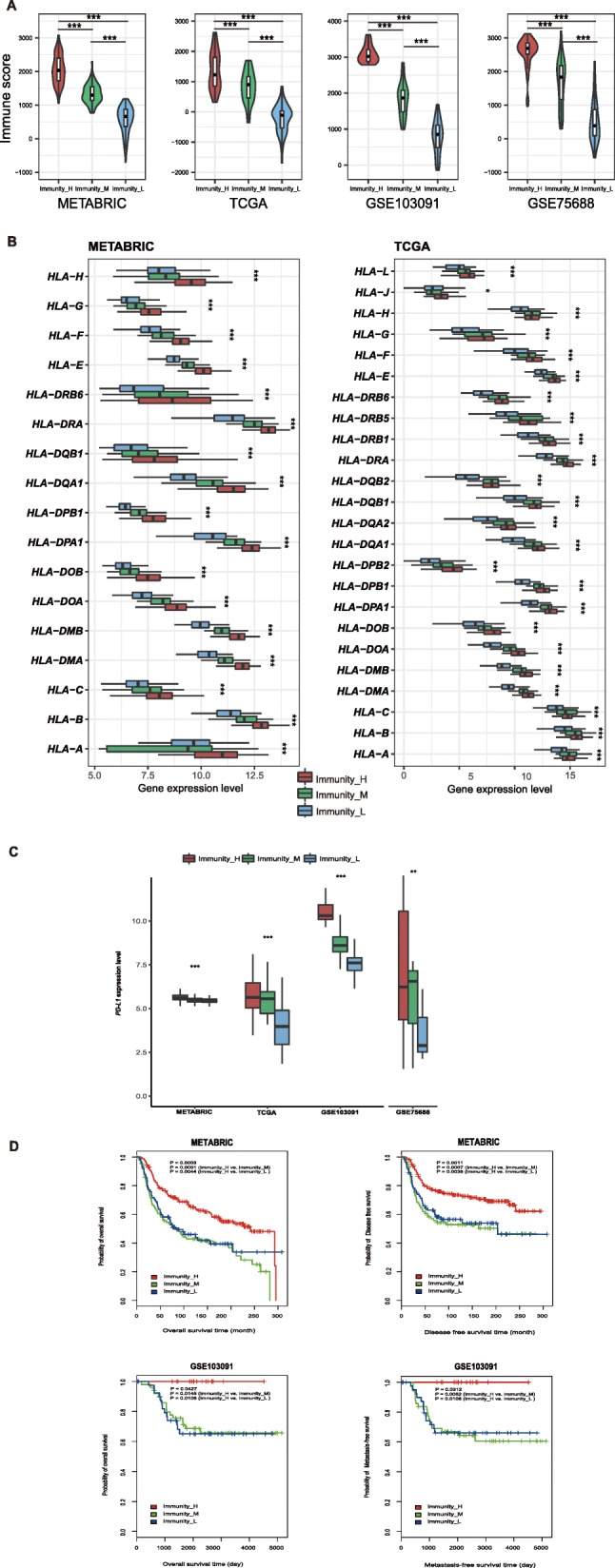
Three TNBC subtypes show differential phenotypes. **a**. Comparison of the immune cell infiltration levels between TNBC subtypes (Mann–Whitney U test). **b**. Comparison of the expression levels of HLA genes between TNBC subtypes (ANOVA test). **c**. Comparison of *PD-L1* expression levels between TNBC subtypes (ANOVA test). **d**. Comparison of survival prognosis between TNBC subtypes (log-rank test). *P < 0.05, **P < 0.01, ***P < 0.001. It also applies to following figures

We analyzed 29 immune-associated gene sets which represented diverse immune cell types, functions, and pathways (Additional file 1: Table S1). We used the ssGSEA score [19, 20] to quantify the activity or enrichment levels of immune cells, functions, or pathways in the cancer samples. On the basis of the ssGSEA scores of the 29 gene sets, we hierarchically clustered TNBC in four BC datasets (METABRIC [31], TCGA [32], GSE75688 [33], and GSE103091 [34]). Interestingly, all four datasets showed similar clustering results, with three clusters being clearly separated (Fig. 1). We defined the three clusters as: Immunity High (Immunity_H), Immunity Medium (Immunity_M), and Immunity Low (Immunity_L). We found that the immune scores were significantly higher in Immunity_H and significantly lower in Immunity_L in all four datasets (Kruskal–Wallis test, *P* < 0.001) (Fig. 2a). Moreover, we found that the percentage of lymphocyte infiltration was significantly higher in Immunity_H and significantly lower in Immunity_L in TCGA based on the pathological slides data (Kruskal–Wallis test, *P* = 0.05). These features directed the classification. In addition, when comparing the tumor purity and stromal score of the three TNBC subtypes, we obtained opposite trends, with tumor purity increasing from Immunity_H to Immunity_L (Immunity_H < Immunity_M < Immunity_L) and stromal score decreasing from Immunity_H to Immunity_L (Immunity_H > Immunity_M > Immunity_L) (Kruskal–Wallis test, *P* < 0.001) (Additional file 2: Figure S1). Collectively, these results suggest that Immunity_H contains the highest number of immune cells and stromal cells, while Immunity_L contains the highest number of tumor cells.

Notably, most HLA genes showed significantly higher expression levels in Immunity_H and significantly lower expression levels in Immunity_L (ANOVA test, *P* < 0.05) (Fig. 2b, Additional file 3: Figure S2A). Moreover, the expression levels of various immune cell subpopulation marker genes [35] were the highest in Immunity_H and the lowest in Immunity_L, such as *CD8A* (cytotoxic T cell), *CD45RO* (memory T cell), *CD20* (B cell), *CXCR5* (Tfh cell), *FOXP3* (Treg), *IL-17* (Th17 cell), *CD1A* (iDC), and *IL3RA* (pDC) (Additional file 3: Figure S2B).

We examined the expression of *PD-L1* (programmed cell death 1 ligand) in the three TNBC subtypes and found that Immunity_H had the highest *PD-L1* expression levels and Immunity_L had the lowest *PD-L1* expression levels (ANOVA test, *P* < 0.05) (Fig. 2c). This suggest that the TNBC subtype Immunity_H might better respond to anti-PD-L1 immunotherapy than the other TNBC subtypes, since *PD-L1* expression tends to be positively associated with immunotherapeutic responsiveness [36].

Survival analyses showed that these TNBC subtypes had distinct clinical outcomes. The Immunity_H subtype likely had a better survival prognosis than the Immunity_M and Immunity_L subtypes, but there was no significant survival difference between the Immunity_M and the Immunity_L subtypes (Fig. 2d). This is consistent with previous studies showing that TNBC with elevated immune activity were associated with more favorable clinical outcomes [4, 12, 34].

### Comparisons of the immunogenomic profiling-based TNBC classification with other TNBC classification methods

**Fig. 3:**
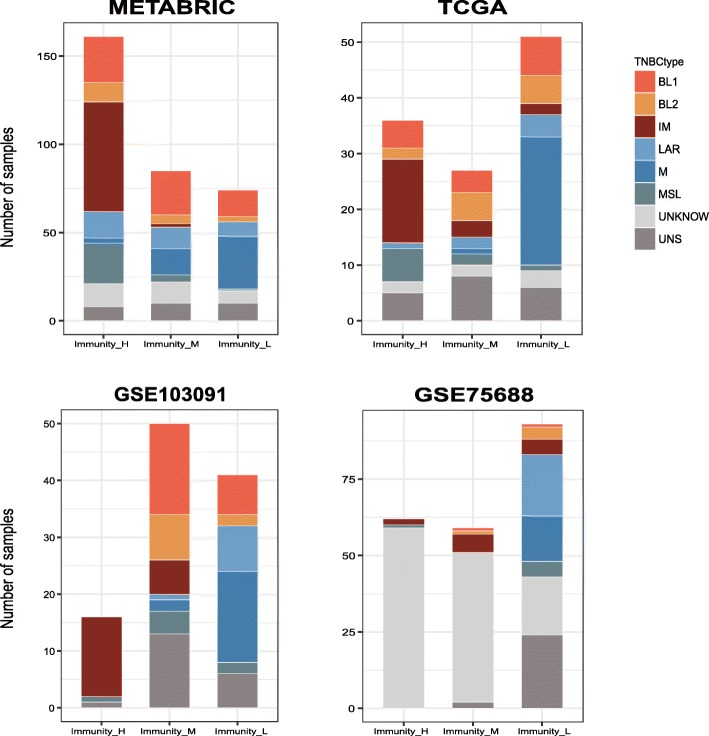
Comparison of the immune signature-based TNBC classification results with the results by TNBCtype shows that Immunity_H is most enriched in IM while Immunity_L is most enriched in M. IM, immunomodulatory; M, mesenchymal

We used the TNBCtype method [2, 37] to classify the four TNBC datasets. We found that the immunomodulatory (IM) subtype of TNBCs was most frequently associated with Immunity_H and least frequently associated with Immunity_L (Fisher’s exact test, *P* < 0.001) (Fig. 3). This is consistent with the enrichment of immune cell processes in the IM subtype [2]. In contrast, the mesenchymal (M) subtype of TNBCs was mostly detected in Immunity_L and least frequently detected in Immunity_H (Fisher’s exact test, *P* < 0.001) (Fig. 3). The M subtype is mainly characterized by pathways involved in cell motility, ECM receptor interaction, and cell differentiation, such as Wnt, ALK, and TGF-β signaling [2]. Our results suggest that the activities of these pathways may be associated with reduced tumor immunity in TNBC.

### Identification of TNBC subtype-specific pathways, gene ontology, and networks

#### Identification of TNBC subtype-specific pathways

**Fig. 4:**
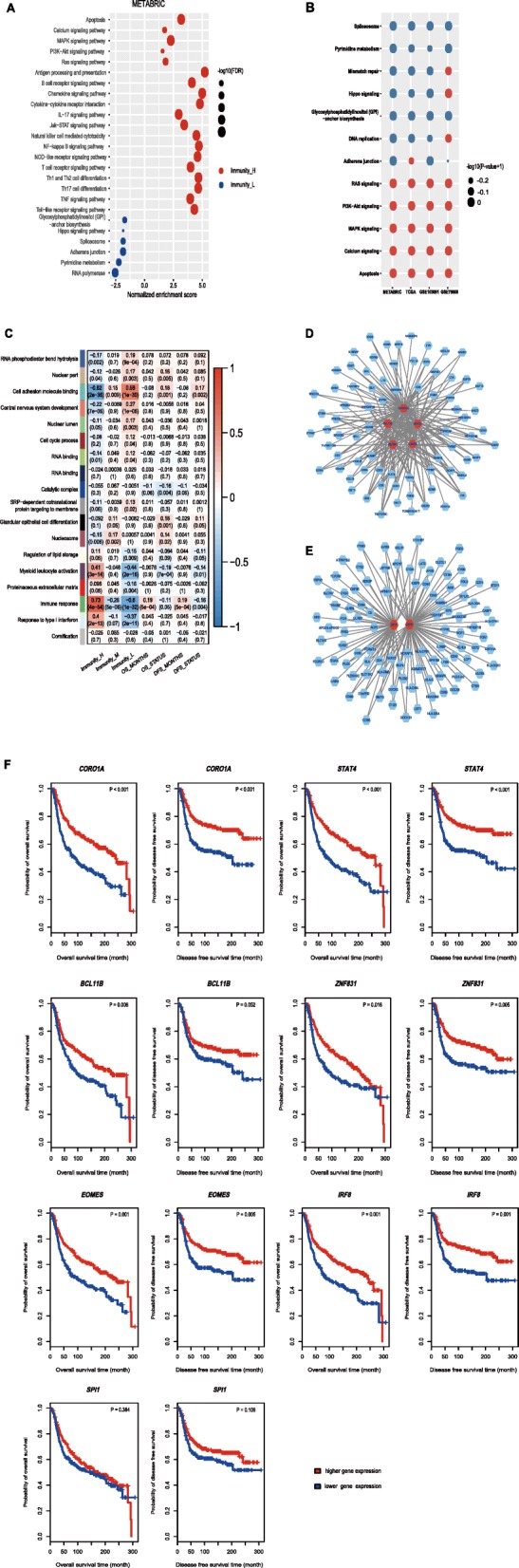
Identification of TNBC subtype-specific pathways, gene ontology, and networks. **a**. KEGG pathways enriched in Immunity_H and Immunity_L. **b**. The cancer-associated pathways upregulated in Immunity_H positively correlated with the immune scores, and the pathways upregulated in Immunity_L negatively correlated with the immune scores in TNBC (Spearman’s correlation test, *P* < 0.05). **c.** Gene modules significantly differentiating TNBC by subtype, survival time, or survival status. **d.** A network significantly active in Immunity_H, centered on five TFs (highlighted in red). **e.** A network significantly active in Immunity_L, centered on two TFs (highlighted in red). **f**. Kaplan–Meier curves showing that the expression of the hub TF genes is positively associated with survival prognosis in TNBC (log-rank test, *P* < 0.05). TF, transcription factor; FDR, false discovery rate

GSEA identified a number of KEGG [25] pathways enriched in Immunity_H and Immunity_L (Fig. 4a, Additional file 4: Figure S3A). Typically, the immune-associated pathways were highly active in Immunity_H and included antigen processing and presentation pathways, B and T cell receptor signaling, chemokine signaling, cytokine–cytokine receptor interactions, IL-17 signaling, Jak–STAT signaling, natural killer cell-mediated cytotoxicity, NF-kappa B signaling, NOD-like receptor signaling, TNF signaling, and Toll-like receptor signaling (Fig. 4a, Additional file 4: Figure S3A). This result confirmed the elevated immune activity in Immunity_H. Besides, we identified various cancer-associated pathways that were hyperactivated in Immunity_H, including apoptosis, calcium signaling, MAPK signaling, PI3K–Akt signaling, and RAS signaling (Fig. 4a, Additional file 4: Figure S3A). This suggests that the activities of these cancer-associated pathways are positively associated with TNBC immunity. In contrast, the TNBC subtype Immunity_L was enriched in pathways related to Hippo signaling, DNA replication, mismatch repair, spliceosome, adherens junctions, pyrimidine metabolism, glycosylphosphatidylinositol (GPI)-anchor biosynthesis, and RNA polymerase (Fig. 4a, Additional file 4: Figure S3A). This indicates that the activities of these pathways could be negatively associated with TNBC immunity. In fact, a previous study has shown that the activities of MAPK and PI3K–Akt cascades positively correlated with the activation of various immune pathways, while the activity of the mismatch repair pathway showed a negative correlation with immune activation in TNBC [12]. Furthermore, we confirmed that all the cancer-associated pathways hyperactivated in Immunity_H were positively associated with the immune scores, whereas the pathways hyperactivated in Immunity_L likely showed a negative correlation (Spearman’s correlation test, *P* < 0.05) (Fig. 4b).

#### Identification of TNBC subtype-specific gene ontology

We performed a weighted gene co-expression network analysis of the METRABRIC dataset by WGCNA [26] and identified a set of gene modules (gene ontology) associated with the highly expressed genes previously determined. We found several gene modules that significantly differentiated TNBC by subtype, survival time, or survival status (Fig. 4c). As expected, the immune response was significantly elevated in Immunity_H (*P* = 4.0*10^− 54^), while was depressed in Immunity_L (*P* = 1.0*10^− 32^). Moreover, a high immune response was associated with a better survival prognosis in TNBC patients (*P* = 5.0*10^− 4^). This finding is in line with the previous observation that the subtype Immunity_H is associated with better clinical outcomes than the other subtypes. Similar results were observed for the TCGA dataset (Additional file 4: Figure S3B). The other two immune-associated gene modules, i.e., myeloid leukocyte activation and response to type I interferon, were also enriched in Immunity_H (*P* = 3.0*10^− 14^ and 2.0*10^− 13^, respectively), and were reduced in Immunity_L (*P* = 2.0*10^− 16^ and 2.0*10^− 11^, respectively). In contrast, cell adhesion molecule (CAMD) binding activity was significantly increased in Immunity_L (*P* = 1.0*10^− 30^) and decreased in Immunity_H (*P* = 2.0*10^− 35^). This suggests that CAMD activity has a strong inverse correlation with tumor immunity in TNBC. Interestingly, CAMD activity correlated with reduced survival (*P =* 0.001 for OS, and *P* = 0.002 for DFS). Cell cycle process was also increased in Immunity_L (*P* = 0.04), suggesting that the cell cycle signature correlates with reduced tumor immunity. This finding is consistent with results from previous studies [38, 39].

#### Identification of TNBC subtype-specific networks

WGCNA generated a gene module (green color, Fig. 4c) that was specifically significant in Immunity_H. We identified 98 hub genes from the gene module, including five transcription factor (TF) genes, i.e., *CORO1A*, *STAT4*, *BCL11B*, *ZNF831*, and *EOMES*. The five TFs interact with each other and form a subnetwork with diverse immune and cancer-related genes that they regulate (Fig. 4d). Typically, *CD247* (the marker gene for a T cell subpopulation) was regulated by all these TFs, and the cytotoxic T cell marker gene *CD8A* was co-regulated by CORO1A, STAT4, and EOMES. *MAP4K1* (Mitogen-Activated Protein Kinase Kinase Kinase Kinase 1), which is involved in multiple immune and cancer-related pathways including B cell receptor signaling, JNK, EGF/EGFR, TGF-β, and MAPK signaling, was also regulated by the five TFs. *CORO1A* encodes a member of the WD repeat protein family which is involved in diverse cellular processes including cell cycle, apoptosis, signal transduction, and gene regulation. The main pathways related to *CORO1A* include cytoskeletal signaling and phagosome function, and its relatedness with immune regulation has been revealed [40, 41]. The association of the other TFs STAT4 [42], BCL11B [43], and EOMES [44] with immunity has been examined, whereas the role of ZNF831 in immune regulation remains unexplored.

WGCNA also generated a gene module (turquoise color, Fig. 4c) that was more enriched in Immunity_L. This module included 112 hub genes, two of which encode the TFs IRF8 and SPI1. A subnetwork of the hub genes centered on *IRF8* and *SPI1*is shown in Fig. 4e. IRF8 (interferon regulatory factor 8) has been shown to play a negative role in immune cell regulation [45]. Thus, the *IRF8*-centered regulatory network may be responsible for the depressed immunity of the TNBC subtype Immunity_L. *SPI1* (Spi-1 proto-oncogene) encodes a transcription factor that activates gene expression during immune cell development. As a result, the deregulation of *SPI1* may affect immunity. In fact, *SPI1* showed significantly lower expression levels in Immunity_L than in Immunity_H (Student’s t test, *P* = 9.1*10^− 28^, fold change > 2). Therefore, the down regulation of *SPI1* may contribute to the decreased immunity of the Immunity_L subtype. The contribution of the *IRF8*- and *SPI1*-centered regulatory network to the depressed immunity of Immunity_L is evidenced by a previous study showing that IRF8 and SPI1 together negatively regulated immune cell differentiation [45].

Interestingly, survival analyses showed that elevated expression levels of these TF genes (except *SPI11*) were consistently associated with better survival prognosis in TNBC (Fig. 4f), suggesting the pivotal role of these TFs in TNBC immunity and prognosis.

### Class prediction of TNBC subtypes based on immunogenomic profiling

**Fig. 5:**
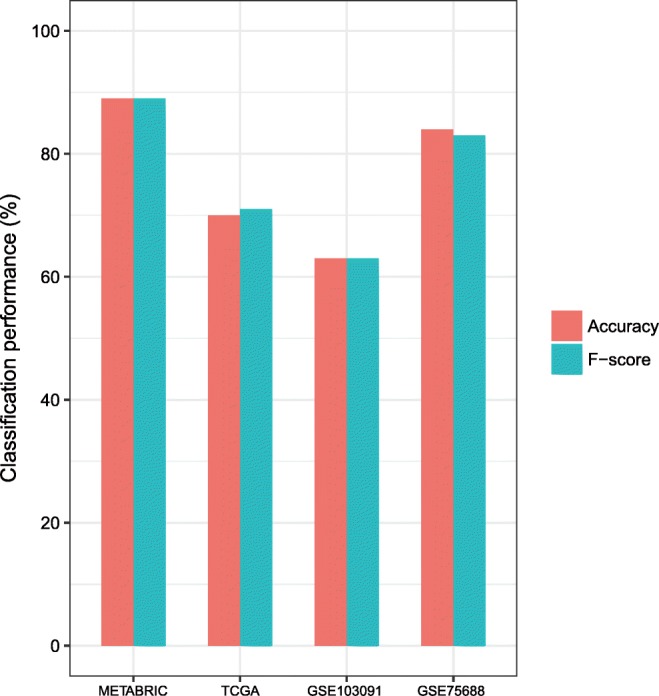
Performance in the classification of TNBC subtypes based on immune signatures. F-score, weighted average of F-scores

We first used 10-fold cross validation (CV) to evaluate the classification performance in METABRIC and then predicted the TNBC subtypes in the other three datasets using the METABRIC dataset as the training set. The 10-fold CV accuracy was 89% in classifying the METABRIC dataset. The classification accuracies were 70, 84, and 63% in TCGA, GSE75688, and GSE103091, respectively. The weighted F-scores in these classifications were 89, 71, 83, and 63% for METABRIC, TCGA, GSE75688, and GSE103091, respectively (Fig. 5). These results demonstrate that the immunogenomic profiling-based classification of TNBC is stable and predictable.

## Discussion

A number of prior studies have identified TNBC subtypes on the basis of genomic profiling [2–4, 34]. However, very few studies have investigated the classification of TNBC specifically based on immune signatures. To fill this knowledge gap, we focused on identifying immune-related TNBC subtypes using immunogenomic profiling. Our results show that TNBC could be classified into three stable subtypes: Immunity High, Immunity Medium, and Immunity Low. Furthermore, we demonstrated that this classification was reproducible and predictable. The Immunity High TNBC subtype was enriched not only in immune signatures, but also in many cancer-associated pathways including apoptosis, calcium signaling, MAPK signaling, PI3K–Akt signaling, and RAS signaling (Fig. 4a). This is in line with our previous study showing that diverse immune signatures positively correlated with the MAPK and PI3K–Akt signaling pathways in TNBC [12]. In contrast, the Immunity Low TNBC subtype was impoverished in immune signatures but enriched in Hippo signaling, DNA replication, mismatch repair, spliceosome, adherens junction, pyrimidine metabolism, glycosylphosphatidylinositol (GPI)-anchor biosynthesis, and RNA polymerase pathways (Fig. 4a). It is rational that the mismatch repair pathway activity was significantly negatively correlated with immune signatures in cancer, since deficient mismatch repair often results in elevated tumor immunity [18]. Interestingly, we found that the Hippo signaling pathway had a significantly negative correlation with immune signatures in TNBC. This observation is in agreement with findings from previous studies showing that the Hippo signaling pathway plays a key role in regulating tumor immunity [46–48]. Deficiency of Hippo pathway components such as kinases LATS1/2 (large tumor suppressor 1 and 2) [46], effector YAP (Yes-associated protein) [47], and transcriptional co-activator TAZ (WW domain-containing transcription regulator 1) [48] could promote anti-tumor immunity. Overall, these results revealed potential positive or negative associations between pathway activities and immune activities in TNBC.

**Fig. 6:**
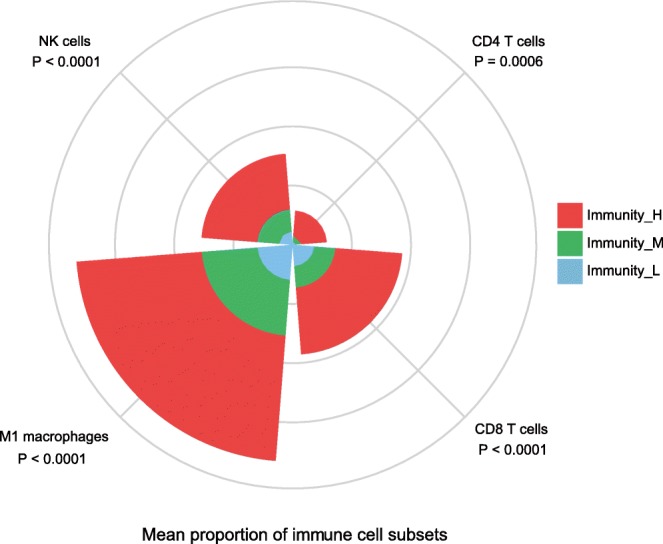
Comparison of the proportions of immune cell subsets between TNBC subtypes. Kruskal-Wallis test, *P* values are shown

Immunity_H had stronger immune cell infiltration and anti-tumor immune activities, e.g., high levels of cytotoxic T cells and B cells infiltration (Fig. 1). When we used CIBERSORT [28] to calculate the proportions of 22 immune cell subsets in TNBC, we found that CD8 T cells, CD4 T cells, NK cells, and M1 macrophages tended to be present in significantly higher numbers in Immunity_H than in Immunity_L (Mann–Whitney U test, *P* < 0.05) (Fig. 6). This analysis again confirmed elevated anti-tumor immune activity in Immunity_H. The high anti-tumor immune activation could explain why Immunity_H had more favorable clinical outcomes compared to the other subtypes (Fig. 2d). In fact, numerous studies have demonstrated that the density of tumor-infiltrating lymphocytes (TILs) is positively associated with survival prognosis in various cancers [35, 49, 50]. Immunity_H more highly expressed most HLA genes, which is indicative of stronger immunogenicity compared to the other subtypes. However, Immunity_H did not show higher TMB or neoantigen load than the other subtypes. This suggests that the differential immunogenicity between the TNBC subtypes cannot be attributed to TMB and neoantigens. In addition, we did not find lower clonal heterogeneity in Immunity_H, as estimated by ABSOLUTE [29], than in the other subtypes, although, in some studies, clonal heterogeneity was shown to have a significant negative correlation with tumor immunity [13, 51]. Interestingly, Immunity_H exhibited more active stem cell-associated biological processes than the other subtypes (Additional file 5: Figure S4), while it showed no significant differences in proliferation and epithelial-to-mesenchymal transition (EMT) processes.

**Fig. 7:**
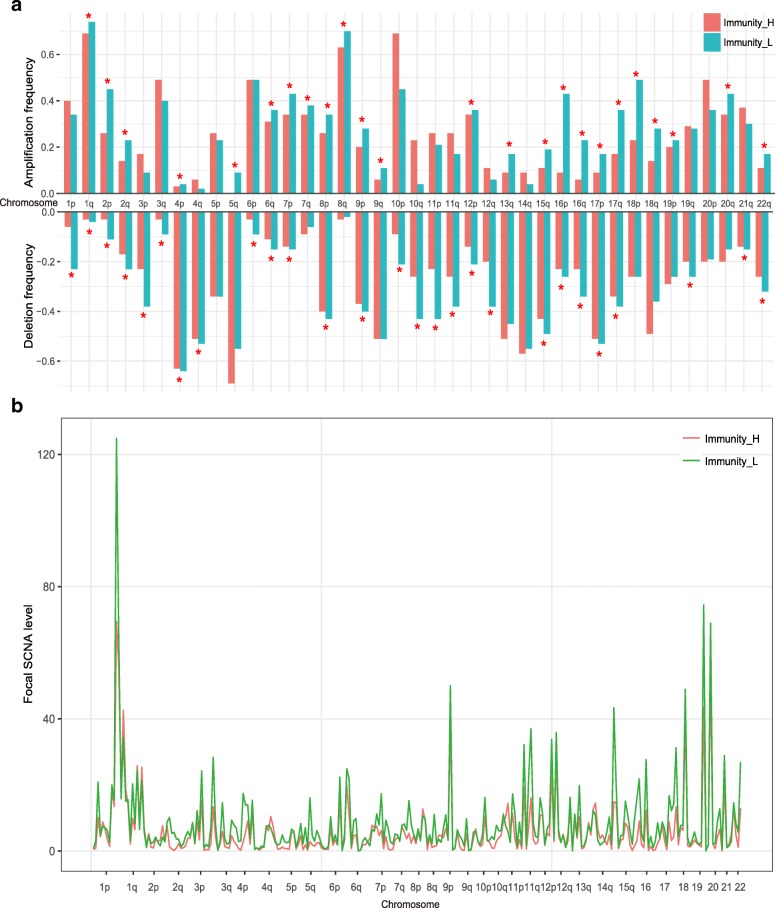
Comparison of the somatic copy number alteration (SCNA) levels between TNBC subtypes**. a.** Comparison of the arm-level SCNAs between Immunity_H and Immunity_L. The red asterisks indicate the chromosome arms in which Immunity_H presents higher amplification or deletion frequency than Immunity_L. **b.** Comparison of the focal SCNA levels between Immunity_H and Immunity_L

Furthermore, we compared SCNA levels between Immunity_H and Immunity_L subtypes. We found that Immunity_H had significantly lower arm-level SCNAs than Immunity_L (Wilcoxon signed-rank test, *P* = 0.04, 0.001, 0.0006 for comparisons of amplification, deletion, and total alteration frequencies, respectively) (Fig. 7a). Moreover, Immunity_H had significantly lower focal SCNA levels than Immunity_L (Mann–Whitney U test, *P* = 0.01, 0.02, 0.01 for comparisons of amplification, deletion, and total alteration levels, respectively) (Fig. 7b). These findings demonstrated that Immunity_H had lower levels of SCNAs compared to Immunity_L, supporting the notion that high tumor aneuploidy correlates with reduced tumor immune infiltration [52].

Currently, immunotherapy for TNBC is an active field of investigation [53], and the stronger immunogenicity exhibited by TNBC compared to other breast cancer subtypes suggests that immunotherapy could be a viable option for TNBC patients [12]. However, some preliminary TNBC immunotherapy clinical trials have not shown significant patients’ improvement (personal communication). Thus, the immune signature-based classification of TNBC may aid the stratification of TNBC patients to identify those responsive to immunotherapy. It is imaginable that patients with an Immunity_H subtype of TNBC would be more likely to respond to anti-PD-1/PD-L1 therapy than patients with other TNBC subtypes, since *PD-L1* is more highly expressed in Immunity_H TNBC, and PD-L1 expression is a predictive biomarker for the response to PD-1/PD-L1-directed immunotherapy [36, 54].

## Conclusions

The identification of TNBC subtypes based on immune signatures has potential clinical implications for TNBC treatment.

## Additional files

Additional file 1: **Table S1.** The 29 immune signatures represented by 29 different gene sets. (XLSX 41 kb)

Additional file 2: **Figure S1.** Comparisons of the stromal content and tumor purity between TNBC subtypes (Mann–Whitney U test). (PDF 368 kb)

Additional file 3: **Figure S2.** Comparisons of the expression levels of immune-related genes between TNBC subtypes. A. Comparisons of the expression levels of HLA genes between TNBC subtypes. B. Comparisons of the expression levels of immune cell subpopulation marker genes between TNBC subtypes. ANOVA test. *P < 0.05, **P < 0.01, ***P < 0.001. (PDF 168 kb)

Additional file 4: **Figure S3.** Identification of TNBC subtype-specific pathways and gene ontology. A. KEGG pathways enriched in Immunity_H and Immunity_L. B. Gene modules significantly differentiating TNBC by subtype, survival time, or survival status. (PDF 164 kb)

Additional file 5: **Figure S4.** Stem cell-associated activity is higher in Immunity_H than in the other subtypes. (PDF 110 kb)

## Abbreviations

aDCs: Activated dendritic cells
BC: Breast cancer
BLIA: Basal-like immune activated
CAMD: Cell adhesion molecule binding
CCR: Cytokine and cytokine receptor
CV: Cross validation
DCs: Dendritic cells
DFS: Disease-free survival
EMT: Epithelial-to-mesenchymal transition
ER: Estrogen receptor
FDR: False discovery rate
GSEA: Gene-set enrichment analysis
HLA: Human leukocyte antigen
HER2: Human epidermal growth factor receptor 2
iDCs: Immature dendritic cells
IM: Immunomodulatory
Immunity_H: Immunity high
Immunity_M: Immunity medium
Immunity_L: Immunity low
M: Mesenchymal
MFS: Metastasis-free survival
MHC: Major histocompatibility complex
NK cells: Natural killer cells
OS: Overall survival
pDCs: Plasmacytoid dendritic cells
PR: Progesterone receptor
RF: Random forest
ssGSEA: Single-sample gene-set enrichment analysis
SCNA: Somatic copy number alteration
TCGA: The Cancer Genome Atlas
TF: Transcription factor
Tfh cells: Follicular helper T cells
Th17 cells: T helper 17 cells
TILs: Tumor-infiltrating lymphocytes
TME: Tumor microenvironment
TNBC: Triple-negative breast cancer
Treg: Regulatory T cells
WGCNA: Weighted gene co-expression network analysis
