# Supplementary figures and images for: Classification of triple-negative breast cancers based on Immunogenomic profiling
Source: J Exp Clin Cancer Res. 2018 Dec 29;37:327. doi: 10.1186/s13046-018-1002-1 (PMC6310928; doi:10.1186/s13046-018-1002-1)

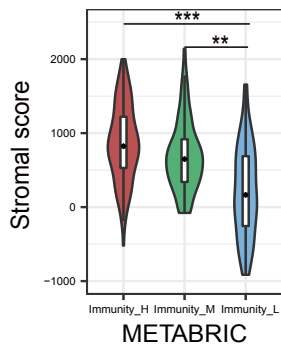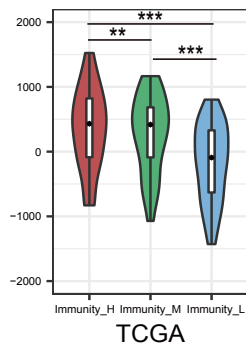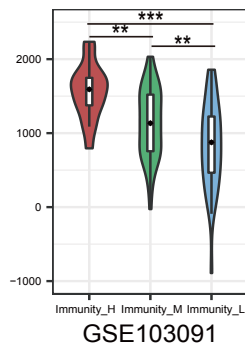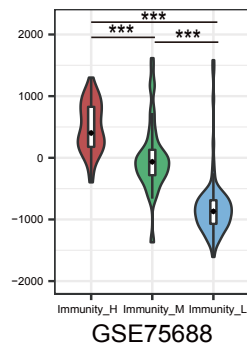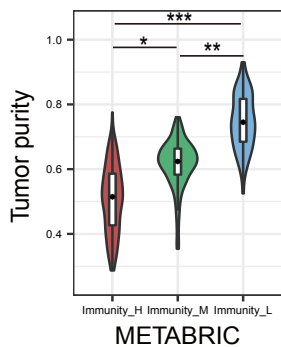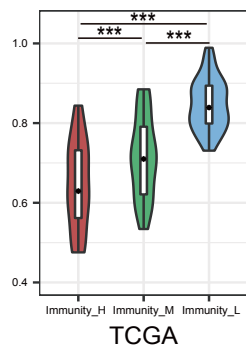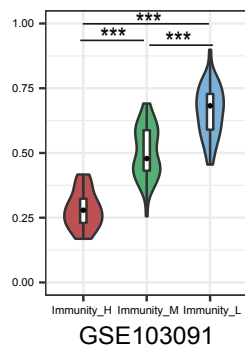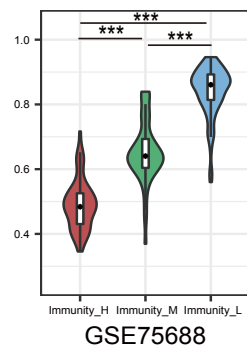

Supplement: Supplementary file 2 — Figure S1. Comparisons of the stromal content and tumor purity between TNBC subtypes (Mann–Whitney U test). (PDF 368 kb) [file 13046_2018_1002_MOESM2_ESM.pdf]

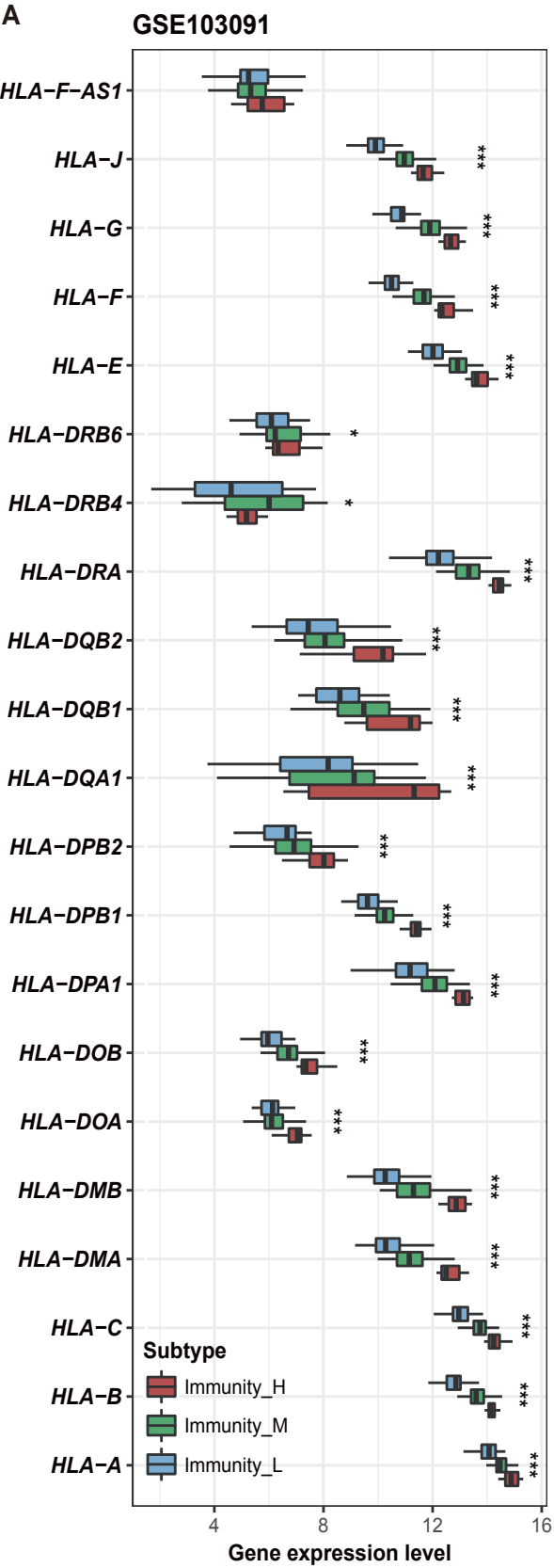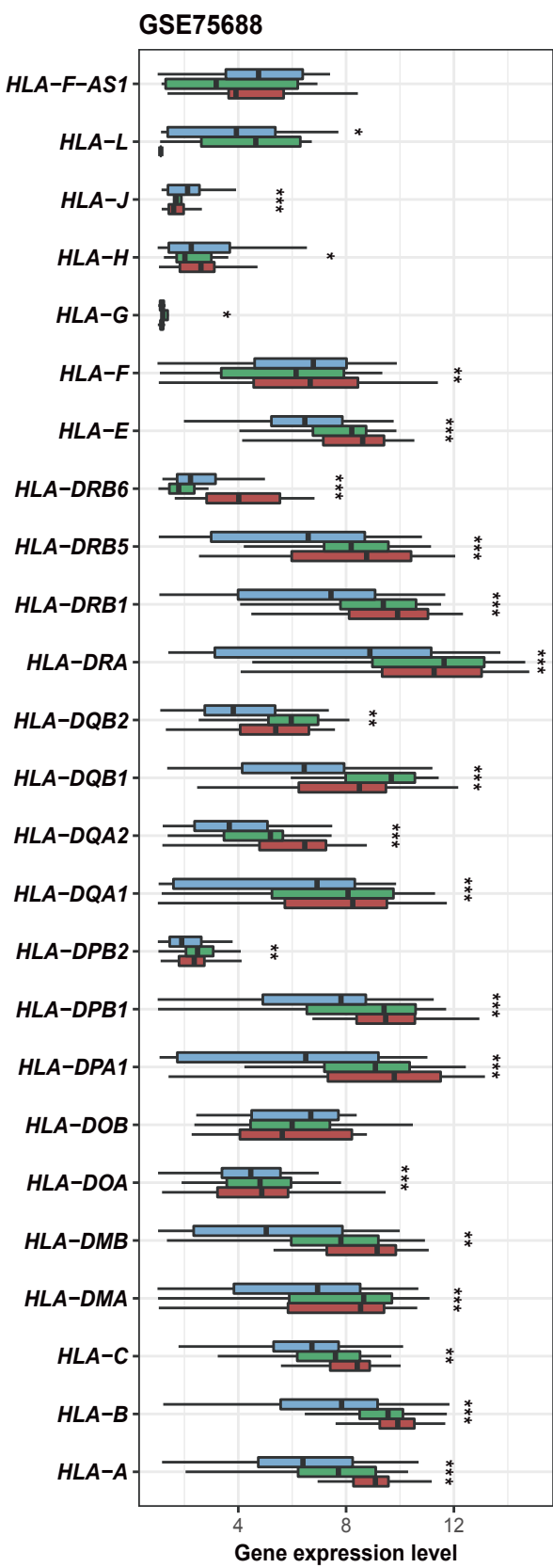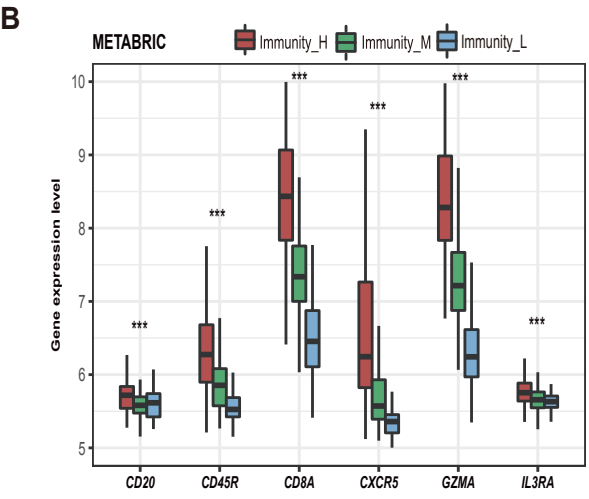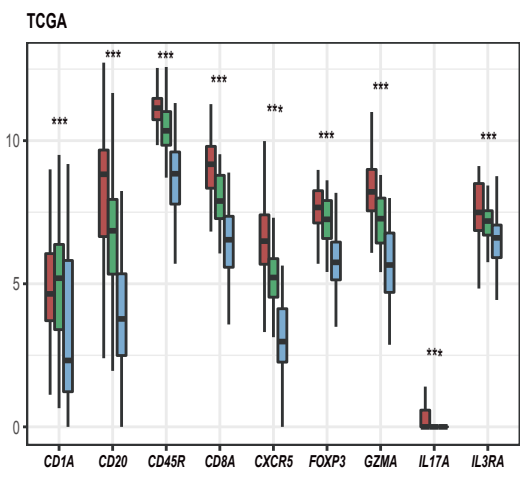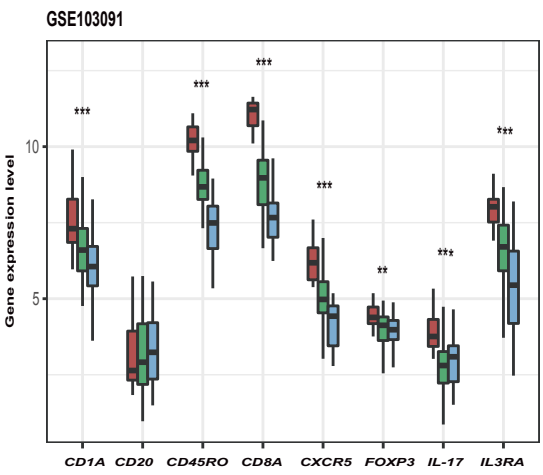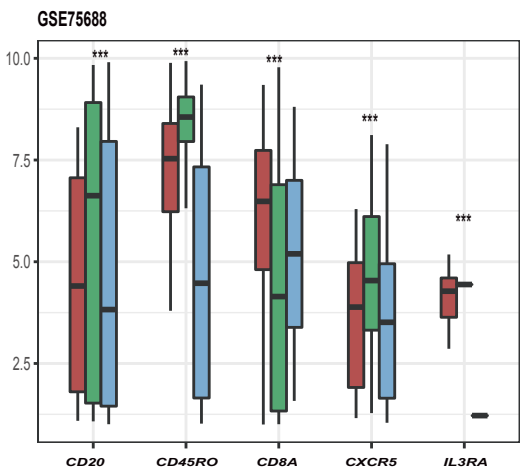

Supplement: Supplementary file 3 — Figure S2. Comparisons of the expression levels of immune-related genes between TNBC subtypes. A. Comparisons of the expression levels of HLA genes between TNBC subtypes. B. Comparisons of the expression levels of immune cell subpopulation marker genes between TNBC subtypes. ANOVA test. *P < 0.05, **P < 0.01, ***P < 0.001. (PDF 168 kb) [file 13046_2018_1002_MOESM3_ESM.pdf]

A

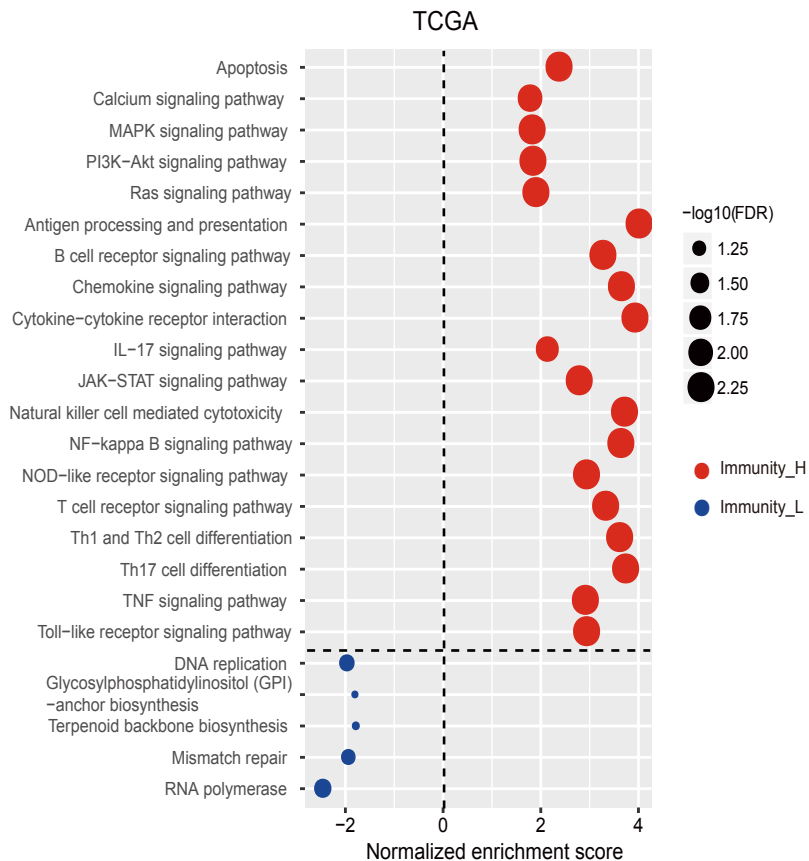

B

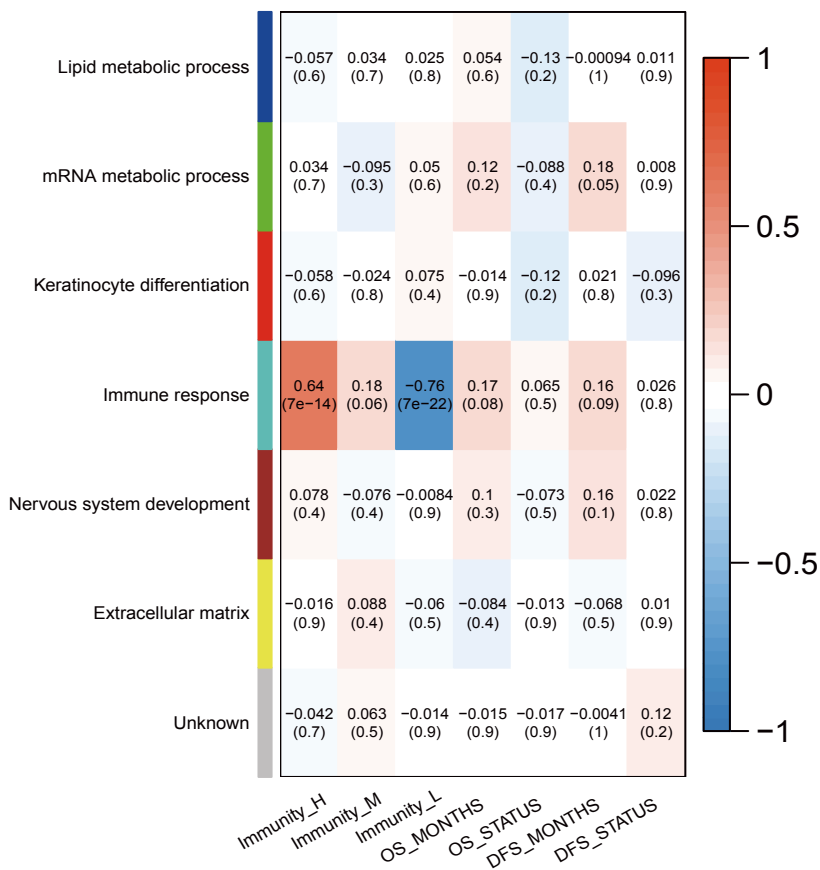

Supplement: Supplementary file 4 — Figure S3. Identification of TNBC subtype-specific pathways and gene ontology. A. KEGG pathways enriched in Immunity_H and Immunity_L. B. Gene modules significantly differentiating TNBC by subtype, survival time, or survival status. (PDF 164 kb) [file 13046_2018_1002_MOESM4_ESM.pdf]

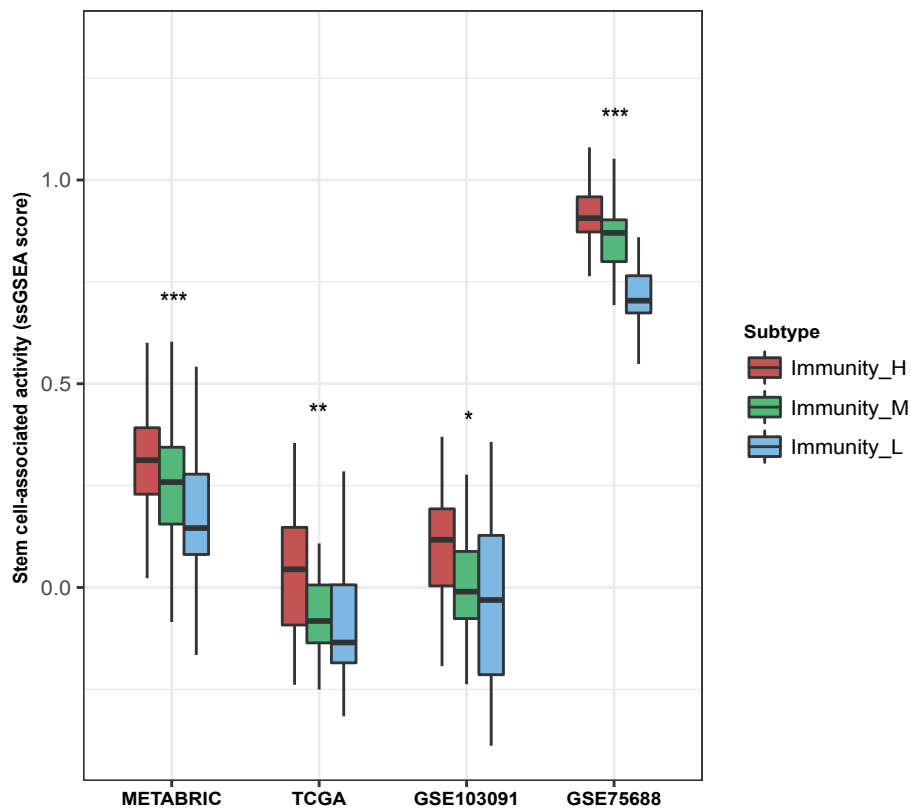

Supplement: Supplementary file 5 — Figure S4. Stem cell-associated activity is higher in Immunity_H than in the other subtypes. (PDF 110 kb) [file 13046_2018_1002_MOESM5_ESM.pdf]
